# Supplementary material for: CDK1 and CDK2 regulate NICD1 turnover and the periodicity of the segmentation clock
Source: EMBO Rep. 2019 Apr 17;20(7):e46436. doi: 10.15252/embr.201846436 (PMC6607002; doi:10.15252/embr.201846436)
Supplement: Supplementary file 1 — Appendix [file EMBR-20-e46436-s001.pdf]

APPENDIX

Appendix Figure S1. Increase in endogenous NICD levels after exposure to small molecule inhibitors is due to increased stability, not increased NICD production. 2

Appendix Figure S2. Point mutations at specific NICD phosphorylated residues do not affect the interaction of NICD with FBXW7.. 4

Appendix Figure S3. Effect of CDK8 and CDK4 depletion on endogenous levels of NICD in HEK293 cells. Effect of CDK1 or CDK2 inhibition on NICD levels and the segmentation clock in embryonic PSM explants.. 6

Mathematical model links NICD regulation and cell cycle 7

Appendix Figure S4. Mathematical model links NICD regulation, segmentation clock and cell cycle.. 10

Mathematical methods 12

Appendix Figure S5. Transition through the cell cycle states and correspondent regions of high CDK2 and CDK 1 activity 16

Appendix Table S1: A table with parameter values. 16

Appendix Table S2: Quantification of Western blots used for inference of parameters *k2* and *k3*. 17

Appendix Figure S1

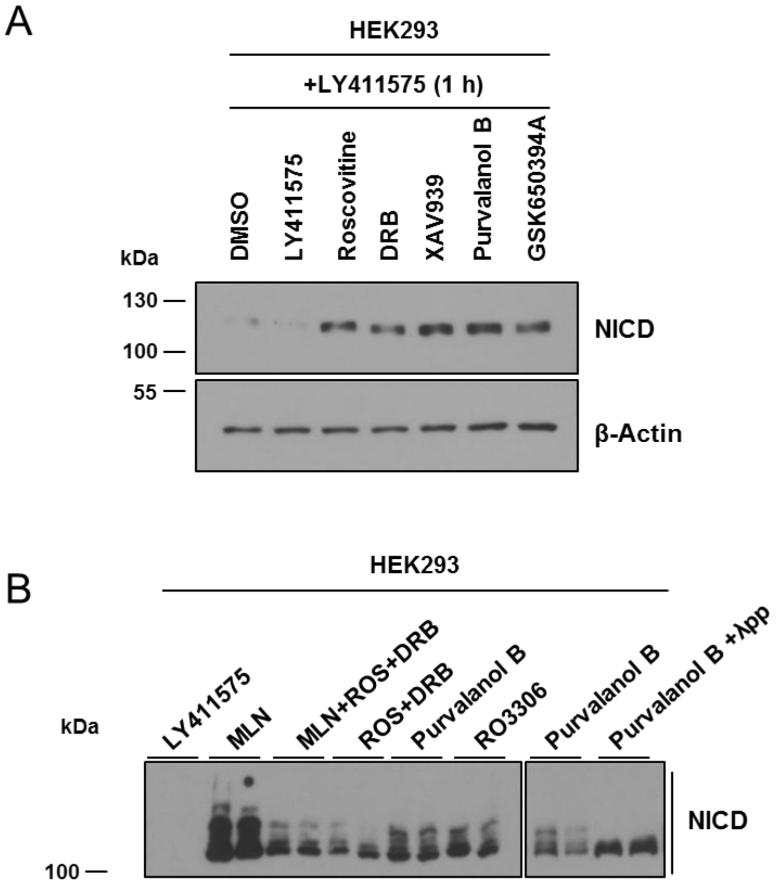

Appendix Figure S1. **Increase in endogenous NICD levels after exposure to small molecule inhibitors is due to increased stability, not increased NICD production**.

A. HEK293 cells were treated for 3 hours with 150 nM of LY411575, 10 µM of Roscovitine, 10 µM of DRB, 10 µM of XAV939, 0.1 µM of Purvalanol B or 10 µM of GSK650394A. DMSO served as vehicle control. 1 hour prior to lysate collection 150 nM of LY411575 was added to DMSO, Roscovitine, DRB, XAV939, Purvalanol B or GSK650394A treated cells to prevent new NICD production. β-Actin served as loading control.
B. HEK293 cells were treated with the same inhibitors as described in Fig1A, in addition to 0.1 µM of Purvalanol B or 10 µM of RO3306. NICD phosphorylation status was analysed by a Phos-tag assay. LY411575 has been used as negative control. The NICD phosphorylation profile varies following treatment with individual small molecule inhibitors as compared to combinations of inhibitors.

Appendix Figure S2

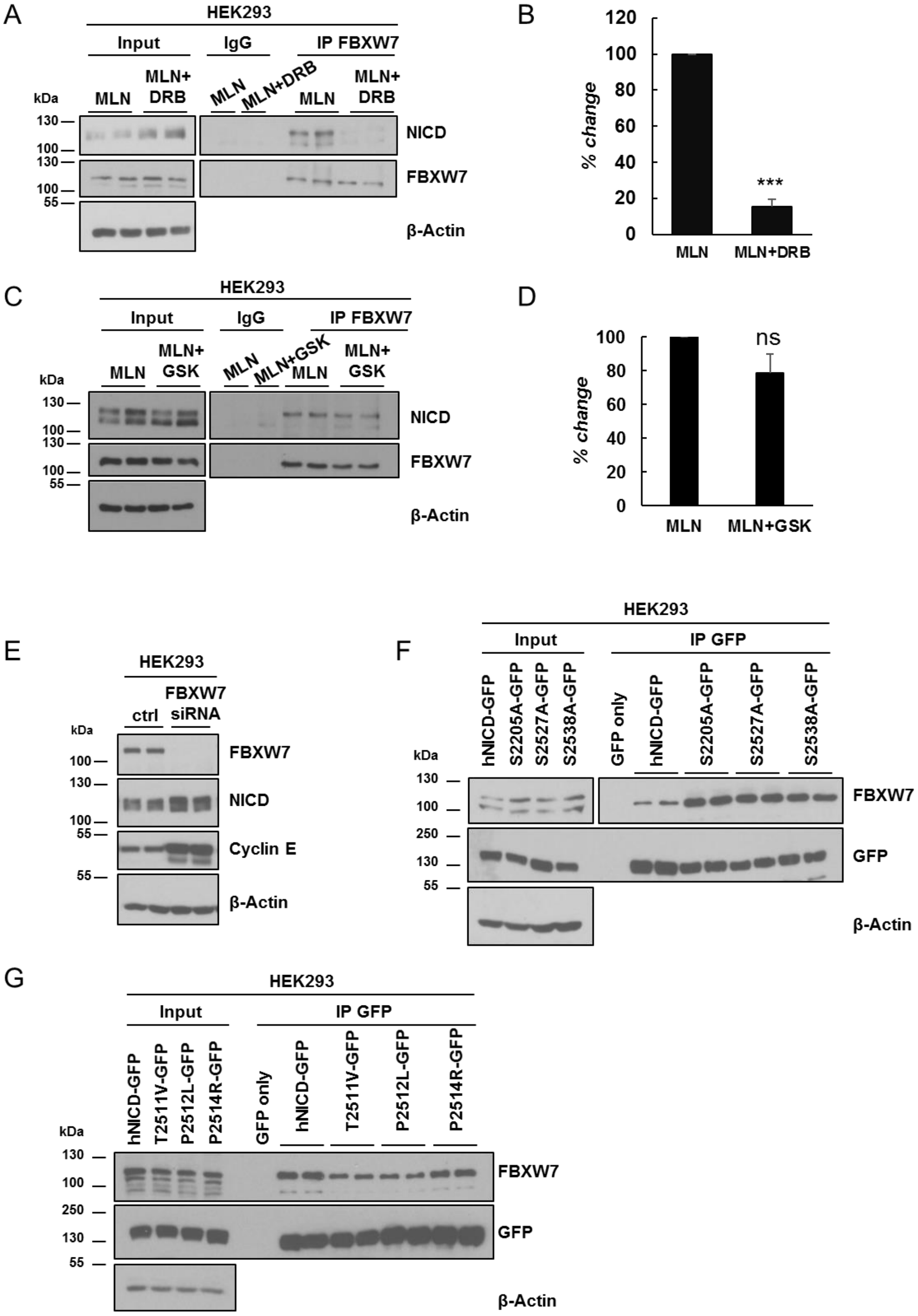

Appendix Figure S2. **Point mutations at specific NICD phosphorylated residues do not affect the interaction of NICD with FBXW7**.

A. Interaction between NICD and FBXW7 is reduced following DRB treatment. 500 µg of HEK293 cell lysates treated with MLN4924 or MLN4924 together with DRB were subjected to immunoprecipitation using FBXW7 antibody, or IgG antibody as negative control, and precipitated material was analysed by western blot using NICD antibody. Western blot with FBXW7 antibody served as loading control for immunoprecipitation efficiency. 10% of cell lysate before immunoprecipitation was used as input control and β-Actin served as loading control.
B. Quantification of the density of western blot bands in (A) performed by ImageJ software. Data are expressed as percentage changes compared to MLN4924 treated samples. All data represent the mean ± SEM from three independent experiments. Student's t-test analysis was performed, with ***$p \leq 0.001$.
C. GSK650394A treatment does not affect the NICD-FBXW7 interaction. 500 µg of HEK293 cell lysates treated with MLN4924 or MLN4924 together with GSK650394A were subjected to immunoprecipitation using FBXW7 antibody, or IgG antibody as negative control, and precipitated material was analysed by western blot using NICD antibody. Western blot with FBXW7 antibody served as loading control for immunoprecipitation efficiency. 10% of cell lysate before immunoprecipitation was used as input control and β-Actin served as loading control.
D. Quantification of the density of western blot bands in (C) performed by ImageJ software. Data are expressed as percentage changes compared to MLN4924 treated samples. All data represent the mean ± SEM from three independent experiments. Student's t-test analysis was performed, with ns= not significant.
E. Inhibiting endogenous FBXW7 modulates endogenous NICD levels. HEK293 cells were transfected with control (scrambled siRNA) or FBXW7 siRNA. Levels of FBXW7, NICD and Cyclin E were determined by western blot. β-Actin served as loading control.

F-G. hNICD-GFP peptides encoding non-phosphorylatable point mutations at S2205, S2527 and S2538 (F) or mutations at T2511, P2512 and P2514 (G) were expressed in HEK293 cells. The exogenously expressed protein was subsequently immunoprecipitated with anti-GFP antibody and precipitated material was analysed by western blot using FBXW7 antibody. Wild-type hNICD-GFP and GFP only vectors were included as positive and negative controls, respectively. Western blot using GFP antibody served as immunoprecipitation efficiency control. β-Actin has been used as loading control.

Appendix Figure S3

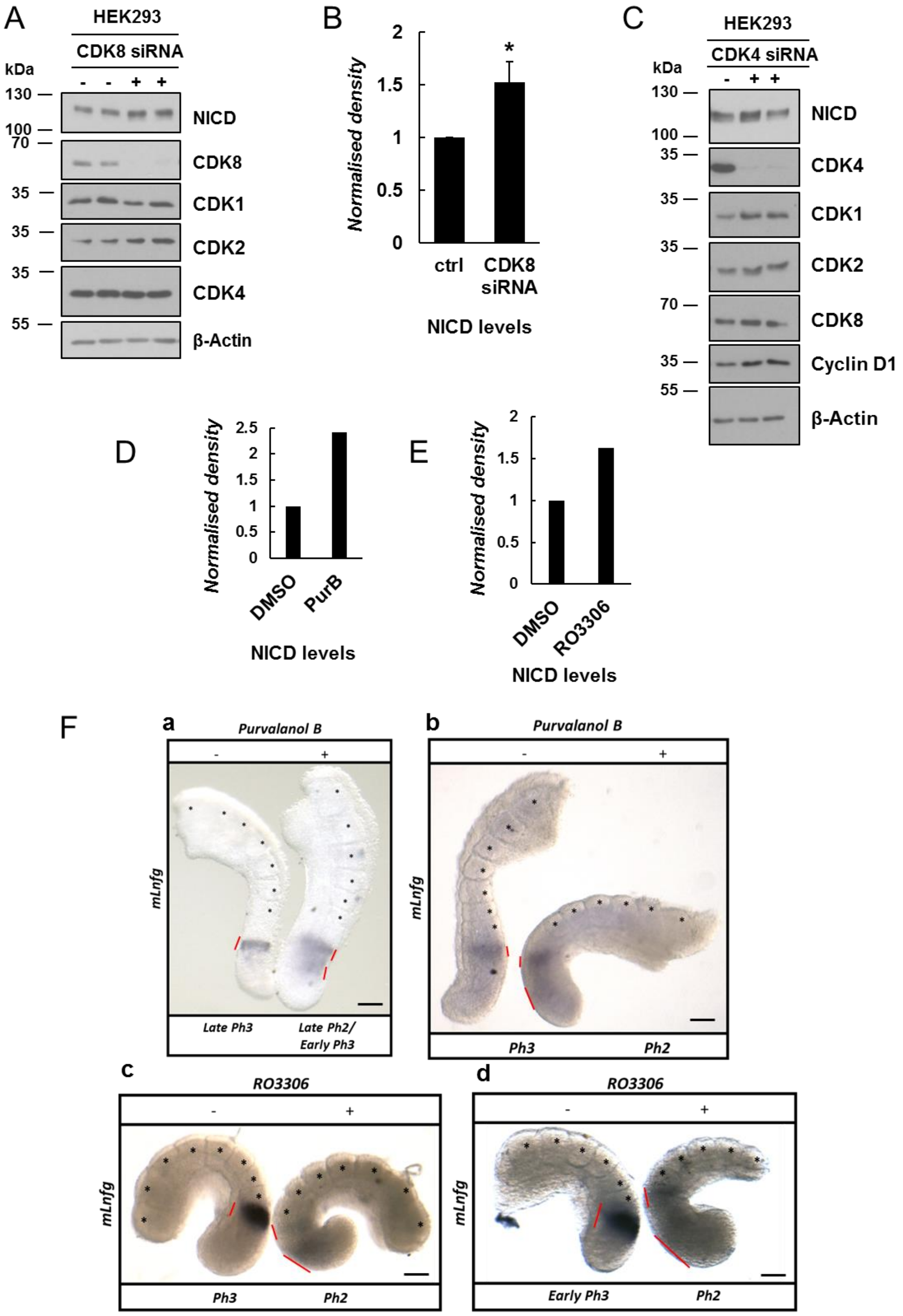

Appendix Figure S3. **Effect of CDK8 and CDK4 depletion on endogenous levels of NICD in HEK293 cells**. **Effect of CDK1 or CDK2 inhibition on NICD levels and the segmentation clock in embryonic PSM explants.**

A. HEK293 cells were cultured for 48h after transfection with plasmids encoding scrambled siRNA or siRNA specific for CDK8, followed by western blot for NICD, CDK8, CDK1, CDK2 and CDK4. β-Actin has been used as loading control.
B. Quantification of the density of western blot bands in (A) performed by ImageJ software. Data are expressed as fold changes compared to control (scrambled siRNA transfected) cell lysate. All data represent the mean ± SEM from three independent experiments. Student's t-test analysis was performed, with *p≤0.05.
C. HEK293 cells were cultured for 48h after transfection with plasmids encoding scrambled siRNA (-) or siRNA specific for CDK4 (+) followed by western blot for NICD, CDK4, CDK1, CDK2, CDK8 and Cyclin D1. β-Actin served as loading control.
D. Quantification of the density of western blot bands in Figure 7C performed by ImageJ software. Data are expressed as fold changes compared to DMSO. All data represent the mean from one pivotal experiment (n=1).
E. Quantification of the density of western blot bands in Figure 7D performed by ImageJ software. Data are expressed as fold changes compared to DMSO. All data represent the mean from one pivotal experiment (n=1).
F. (a-b) Bissected E10.5 mouse PSM explants were cultured in the absence (-) or presence (+) of 1 µM of Purvalanol B for 4 hours and then analysed by *in situ* hybridization for *mLfng* mRNA expression. Purvalanol B treated explants have one less somite than the control explant and the treated explant is in phase 2 of the oscillation cycle and is thus delayed with respect to the control half in phase 3 of the oscillation cycle of dynamic *mLfng* mRNA expression (n=18). Scale bar is 100 µM.

   (c-d) Bissected E10.5 mouse PSM explants were cultured in the absence (-) or presence (+) of 10 µM of RO-3306 for 4 hours and then analysed by *in situ* hybridization for *mLfng* mRNA expression. RO-3306 treated explants have one less somite than the control explant and the treated explant is in phase 2 of the oscillation cycle and is thus delayed with respect to the control half in phase 3 of the oscillation cycle of dynamic *mLfng* mRNA expression (n=10). Scale bar is 100 µM.

Mathematical model links NICD regulation and cell cycle

To understand how our findings on the molecular details of NICD regulation in individual cells give rise to tissue-scale delay of the segmentation clock, we first developed a mathematical model of NICD production and degradation in HEK293 cells. The variables in the model define the position of a cell in the cell cycle, the amount of NICD and the amount of phosphorylated NICD (pNICD) at time t. The model enables us to connect assumptions about molecular processes in individual cells with experiments performed on a large population of cells.

To describe cell-cycle dependent NICD degradation in both cell cycle synchronised and desynchronised experiments, the cell cycle is represented by a linear K-compartment model (**Appendix S4A**) in which progression between successive compartments occurs at constant rate k. G1, S, G2 and M phases of the cell cycle are represented by N_{G1}, N_S, N_{G2} and N_M compartments, respectively, with $K = N_{G1} + N_S + N_{G2} + N_M$. It has previously been shown that of the order of tens of compartments are necessary for such a model to yield reasonable approximation to experimental measurements of cell cycle distribution times [1]. Whilst this model of the cell cycle does not describe the molecular machinery underpinning the cell cycle, it has few parameters and provides a natural framework to simulate cell cycle block experiments.

It is assumed that CDKs that phosphorylate NICD, resulting in its interaction with FBXW7 and subsequent degradation, are active in some m of the cell cycle states. We assume that: (i) NICD exists in non-phosphorylated and phosphorylated forms; (ii) NICD is produced at constant rate k_1; (iii) both forms of NICD get degraded at background rate k_7; (iv) NICD gets phosphorylated by CDKs at rate k_3; (v) pNICD degrades at rate k_6 with $k_6 > k_7$; and (vi) dephosphorylation of NICD occurs at rate k_2. We consider the pharmacological perturbations as follows: LY411575 treatment experiments, in which NICD production is inhibited, are simulated by setting $k_1 = 0$; Purvalanol B/Roscovitine treatment experiments, which target only CDK2, reduce the number of compartments where CDKs are active by a factor of two; and MLN4924 treatment, which inhibits FBXW7 mediated degradation of NICD, are simulated by

removing the fast mode of decay (k_6 = 0).

By averaging cellular descriptions of NICD and the cell cycle over a given cell population, we derive equations describing the average levels of total NICD (i.e. as measured in a western blot experiment, see Methods). Upon release from double thymidine block, cells are synchronised in the cell cycle and there are relatively high levels of CDK activity at different times post-release. As pNICD degrades faster, high levels of CDK results in lower levels of NICD (see **Appendix S4** and **Figures 4A-D, 4F-I, 5B, Figure 7C-D, Appendix S3D-E**). We find the model qualitatively fits the experimental observations if NICD is phosphorylated in two separate time windows post-release. We propose that these windows correspond to activity of CDK2 (G1/S phase) and CDK1 (M phase) (**Appendix S4A** and **S4B**). In a situation where the cell cycle is desynchronised, the population-averaged phosphorylation rate (mk_3/K, where m/K is the fraction of the cell cycle where CDKs are active) is a fraction of the true phosphorylation rate. The model can reproduce western blot results from DMSO and MLN4924 experiments (**Appendix S4C**), allowing inference of the parameters k_2 and k_3. Notably, the simulated Purvalanol B/Roscovitine treatment experiment presented in **Appendix S4C** is a prediction that is validated by the experimental data (**Figures 1B**, **1D** and **4G**).

To explore how CDK inhibition results in delay of the segmentation clock, we introduce an additional variable representing phase of the somitogenesis clock and assume that the caudal PSM behaves like a population of phase-coupled oscillators. In Wiederman *et al*. we proposed a model that showed how competition between activators and inhibitors of clock gene transcription could lead to the oscillator period being an increasing function of NICD half-life [2]. Here, we impose this assumption without explicitly describing the molecular circuitry. We assume that each cellular oscillator has a natural frequency $\omega_i(t)$ that is a function of cell cycle position; when CDKs are active the baseline clock frequency, ω_0, is increased by an acceleration factor r (**Appendix S4D**). As the cell cycle is asynchronous in the PSM, it is therefore comprised of a population of oscillators with frequencies ω_0 or ωr_0. Given sufficiently strong coupling, a population of such oscillators yields synchronous oscillations with a period

that is an average of the individual oscillator periods (**Appendix S4E-F**). Upon CDK inhibition, the relative number of faster oscillators is reduced hence the average period decreases (**Appendix S4E**). Thus, the cell cycle somitogenesis coupled model provides a description of how CDK-mediated phosphorylation of NICD can result in the observed phenotype in PSM tissue (**Figure 7**).

Appendix Figure S4

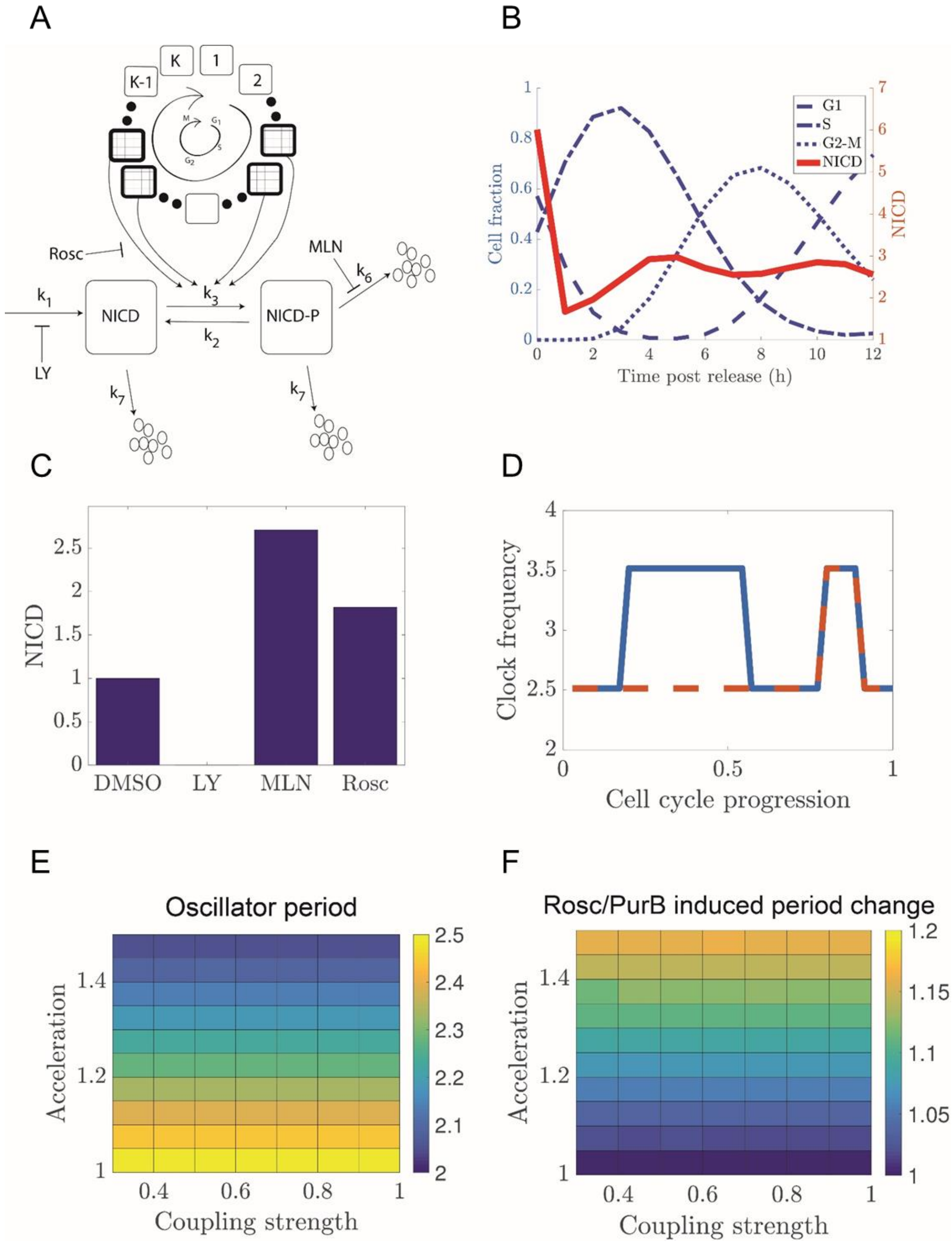

Appendix Figure S4. Mathematical model links NICD regulation, segmentation clock and cell cycle.

A. A schematic illustration of NICD production, phosphorylation and cell-cycle coupled degradation. The cell cycle is represented by clockwise progression through a *K* state model. NICD phosphorylation occurs at two different stages of the cell cycle (patterned

boxes).

B. Levels of NICD and cell cycle phase distribution are plotted against time post-thymidine release.

C. Steady-state levels of NICD are plotted for simulated control, LY411575, MLN4924 and Roscovitine treatments.

D. Simulating a population of coupled PSM oscillators with cell cycle modulated frequencies. The clock frequency in a single cell is plotted against cell cycle position for normal (solid line) and CDK2 inhibited (dashed line) cells.

E. The emergent population-scale oscillator period is plotted against coupling strength and CDK acceleration factor. Period change as a result of CDK inhibition (Roscovitine/Purvalanol B versus control) is plotted against coupling strength and CDK-mediated acceleration rate.

Mathematical methods

NICD - Cell cycle model

To account for the experimental observations of NICD degradation in cell lines, we develop a cell cycle-dependent model of NICD production and degradation (see **Appendix S4A**). The position of the jth cell in the cell cycle is represented by a set of K discrete state variables, X_{ij} (t), defined such that

$$\sum_{i=1}^{K} X_{ij} = 1, \qquad X_{ij} \in \{0,1\}.$$

It is assumed that cells transition sequentially between states at rate r_1 and that mitosis is followed by G1 entry. The averaged fraction of cells in state i, given by

$$\langle X_i \rangle = \frac{1}{n}\sum_{j} X_{ij},$$

where n is the number of cells in the experiment, satisfy

$$\frac{d\langle X_1 \rangle}{dt} = r_1(\langle X_k \rangle - \langle X_f \rangle),$$

$$\frac{d\langle X_j \rangle}{dt} = r_1\big(X_{j-1} - \langle X_j \rangle\big) \quad j = 2,..,K$$

We note that Yates and colleagues [1] propose a similar model and show that relatively large number of compartments (K=25) compartments can be needed to reproduce experimentally observed variances in cell cycle distribution times.

Letting $\langle X \rangle(t)$ represent the population average fraction of cells where CDKs are active, then

$$\langle X \rangle(t) = \sum_{j \in M} \langle X_j \rangle,$$

where M, the set of CDK active states, has m members.

We make the following assumptions about NICD: (i) NICD exists in two states (nonphosphorylated and phosphorylated); (ii) NICD is produced and degraded at constant rates *k1* and *k7*, respectively; (iii) NICD is phosphorylated by CDK1 and/or CDK2 at rate *k3*; (iv) phosphorylated NICD has a larger degradation rate, *k6*, than the background rate, *k7*; and

(v) dephosphorylation occurs at rate *k2*. Letting $N_{1j}(t)$ and $N_{2j}(t)$ represent the amount of NICD and phospho-NICD in the jth cell at time *t*, the population averages are defined to be

$$\langle N_1\rangle = \frac{1}{n}\sum_j N_{1j} \quad \text{and} \quad \langle N_2\rangle = \frac{1}{n}\sum_j N_{2j}$$

and satisfy

$$\frac{d\langle N_1\rangle(t)}{dt} = k_1 + k_2\langle N_2\rangle - \langle k_3\sum_{j\in C} X_j N_1\rangle - k_7\langle N_1\rangle$$

$$\frac{d\langle N_2\rangle(t)}{dt} = k_3\langle\sum_{j\in C} X_j N_1\rangle - k_2\langle N_2\rangle - (k_6 + k_7)\langle N_2\rangle$$

As the cell cycle variables are uncoupled from the NICD dynamics, in a typical cell line experiment each of the X_j's will tend to the equilibrium value

$$X_j = \frac{1}{K},$$

and the fraction of CDK active cells is

$$X = \frac{m}{K}.$$

Hence equations

$$\frac{d\langle N_2\rangle(t)}{dt} = k_3\langle\sum_{j\in C} X_j N_1\rangle - k_2\langle N_2\rangle - (k_6 + k_7)\langle N_2\rangle$$

reduce to

$$\frac{d\langle N_1\rangle(t)}{dt} = k_1 + k_2\langle N_2\rangle - k_3\frac{m}{K}\langle\sum_{j\in C} X_j N_1\rangle - k_7\langle N_1\rangle$$

$$\frac{d\langle N_2\rangle(t)}{dt} = k_3\frac{m}{K}\langle N_1\rangle - k_2\langle N_2\rangle - (k_6 + k_7)\langle N_2\rangle$$

To compute NICD levels equations $\frac{d\langle N_2\rangle(t)}{dt} = k_3\frac{m}{K}\langle N_1\rangle - k_2\langle N_2\rangle - (k_6 + k_7)\langle N_2\rangle$ are solved to steady state (N_1^*, N_2^*) and the total amount of NICD (**Appendix S4B** and **S4C**) is

$$N^* = N_1^* + N_2^*.$$

LY treatment experiments, in which the production of NICD is reduced owing to gamma-secretase inhibition, are simulated by setting *k1*=0. As PurB/Roscovitine is assumed to inhibit CDK2, the effect in the cell cycle synchronised experiments is to reduce the parameter *m*. As MLN treatment inhibits FBXW7-mediated degradation, it is simulated by removing the fast decay mode (i.e. *k6*=0).

Now consider a situation in which cells are synchronised as a result of double thymidine block. At the point of release the cell cycle distribution is no longer at equilibrium, rather the initial conditions are

$$X_i(0) = \begin{cases} \frac{1}{s} & i \in S \\ 0 & \text{otherwise} \end{cases}$$

where *S* is a set of states where cells get paused as a result of double thymidine block. After the cells are released from the cell cycle block, there are peaks of CDK activity (see **Appendix S4B**) and therefore dynamic levels of NICD.

Somitogenesis clock and the cell cycle

To explore the coupling between cell cycle mediated NICD degradation and the somitogenesis clock oscillator in PSM tissue, we introduce an additional set of variables, $\theta_i(t)$, that represent the position of the ith cell in the somitogenesis clock cycle.

As PSM oscillators are coupled via Delta-Notch signalling we consider a phase coupled oscillator model [3] [4] [5] of the caudal PSM given

$$\frac{d\theta_i}{dt} = \omega_i(X_1, X_2, .., X_K) + A \sum_j \sin(\theta_j - \theta_i),$$

where ω_i, the natural frequency of the ith cell, is a functional of cell cycle position, the sum is taken over all oscillators and the coupling function represents the effect of Delta-Notch signalling in the caudal PSM.

In a previous model [2] we demonstrated how competition between NICD and Hes7 could yield a phenotype in which increased levels of NICD are correlated with a longer clock period; here we impose this assumption in the phase coupled oscillator model by assuming that the clock frequency is inversely correlated with levels of NICD (**Appendix S4E**). Hence, as the cell progresses through the cell cycle, basal levels of NICD fluctuate and modify the natural frequency. We assume that

$$\omega_i(X_1, X_2, \dots, X_K) = \begin{cases} r\omega_0 & \text{CDK Active} \\ \omega_0 & \text{otherwise} \end{cases}$$

where the parameter *r* represents the degree of acceleration of the clock when CDK is active. The last two equations represent a mathematical model of coupled PSM oscillators whose frequencies are modulated by the cell cycle position. The model allows us to explore whether there is an emergent population-scale period and how it is affected by perturbing the cell cycle. To do this we simulate a population of *N* cells and find that given adequate oscillator coupling the oscillators synchronise to an emergent frequency, which is an average over the individual oscillator frequencies

Parameter inference

Cell cycle

The parameters used in the model are presented in **Appendix Table S1**.

To recapitulate the cell cycle phase distributions presented in **Figure 5** of the main text we use a 35 state model. The transition rate between compartments, r_1, is defined such that the mean time for a cell to progress from state 1 to is the cell cycle period, T_C=12h. Based upon the data in **Figure 5** in the main text we assume the cell cycle times presented in **Appendix Table S1** (see **Appendix S5**).

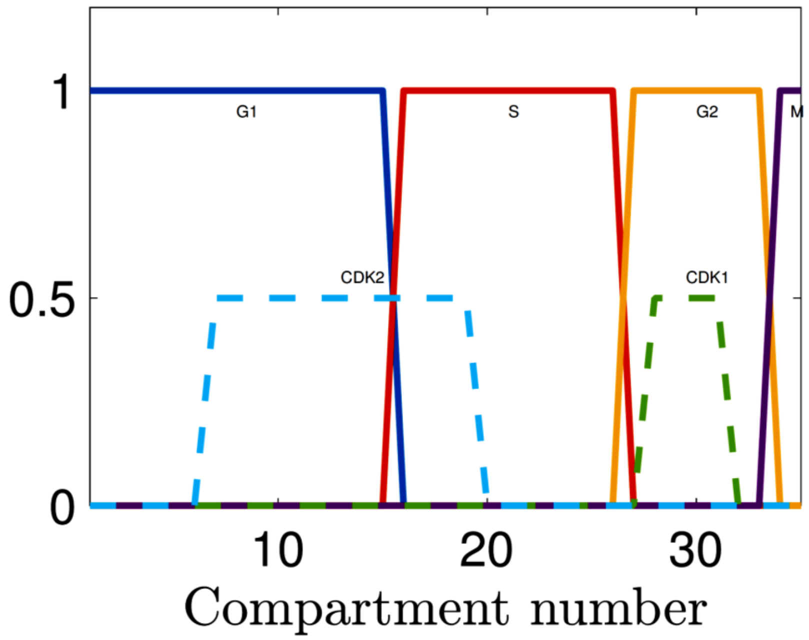

Appendix Figure S5. Transition through the cell cycle states (solid lines) and correspondent regions of high CDK2 and CDK 1 activity (dashed lines).

PARAMETER	***VALUE***	***UNIT***	***DESCRIPTION***
K	35		Number of compartments in cell cycle model
TC	12.0	h	Cell cycle period
R_1	$\ln(2)/KTC$	h^{-1}	Transfer rate between cell cycle states
K_1	10.0	h^{-1}	Rate of NICD production
K_6	12.0	h^{-1}	Fbxw7 mediated NICD degradation rate
K_7	1.6	h^{-1}	Background NICD degradation rate
K_2	3.1	h^{-1}	NICD dephosphorylation rate
K_3	10.0	h^{-1}	NICD phosphorylation rate
ω_0	2.5	h^{-1}	somitogenesis clock frequency
A	[0,2.0]	h^{-1}	Oscillator coupling strength
R	[1,1.5]	h^{-1}	Somitogenesis clock acceleration factor
T_{G1}	4	h	G1 duration
T_S	3	h	S duration
T_{G2}	2	h	G2 duration
T_M	1	h	M duration
CDK2ON	[0.2,0.55]	Nondim	CDK2 active
CDK1ON	[0.8,0.9]	Nondim	CDK1 active
S	[0.4, 0.75]	Nondim	Thymidine block

Appendix Table S1: A table with parameter values.

NICD modelling

The parameter k_1 represents the number of molecules produced per cell per hour. The fast and slow degradation rates are values inferred from [2]. To determine the parameters k_2 and k_3 we use the DMSO and MLN treatment Blot data presented in **Figure 1** of the main text. The measured normalised levels of non-phosphorylated and phosphorylated forms of NICD in different conditions are given in **Appendix Table S2**. A least squares minimisation algorithm is used to minimise the error between the model and the data in **Appendix Table S2** for the control and MLN datasets. Hence, values for the parameters k_2 and k_3 are inferred.

Experiment	***NICD***	***pNICD***
Control	0.9	0.1
MLN	1.5	2.0
Ros	1.3	0.4

Appendix Table S2: Quantification of Western blots used for inference of parameters *k2* and *k3*.

Somitogenesis clock

The somitogenesis clock period in the mouse PSM is approximately two hours. As we do not have direct measurements of coupling, strength or acceleration factor we simulate model behaviour over a range the specified parameter ranges.

1. Yates, C.A., M.J. Ford, and R.L. Mort, *A Multi-stage Representation of Cell Proliferation as a Markov Process.* Bull Math Biol, 2017. **79**(12): p. 2905-2928.
2. Wiedermann, G., R.A. Bone, J.C. Silva, M. Bjorklund, P.J. Murray, and J.K. Dale, *A balance of positive and negative regulators determines the pace of the segmentation clock.* Elife, 2015. **4**: p. e05842.
3. Murray, P.J., P.K. Maini, and R.E. Baker, *The clock and wavefront model revisited.* J Theor Biol, 2011. **283**(1): p. 227-38.
4. Murray, P.J., P.K. Maini, and R.E. Baker, *Modelling Delta-Notch perturbations during zebrafish somitogenesis.* Dev Biol, 2013. **373**(2): p. 407-21.
5. Morelli, L.G., S. Ares, L. Herrgen, C. Schroter, F. Julicher, and A.C. Oates, *Delayed coupling theory of vertebrate segmentation.* HFSP J, 2009. **3**(1): p. 55-66.
