# Supplementary material for: CDK1 and CDK2 regulate NICD1 turnover and the periodicity of the segmentation clock
Source: EMBO Rep. 2019 Apr 17;20(7):e46436. doi: 10.15252/embr.201846436 (PMC6607002; doi:10.15252/embr.201846436)
Supplement: Supplementary file 2 — Expanded View Figures PDF [file EMBR-20-e46436-s002.pdf]

Expanded View Figures

Figure EV1. Exposure to Roscovitine, DRB or XAV9393 leads to increased levels of NICD in IMR90 and mES cells.

A IMR90 cells were treated for 3 h with 150 nM of LY411575, 1 μM of MLN4924, 10 μM of Roscovitine, 10 μM DRB or 10 μM XAV939. DMSO served as vehicle control. Western blot analysis reveals that NICD levels were increased upon treatment with Roscovitine, DRB, XAV939 and MLN4924. NICD is undetectable following LY411575 treatment. β-Actin served as loading control.

B Quantification of the density of Western blot bands in (A) using ImageJ software. Data are expressed as fold changes compared to DMSO. All data represent the mean ± SEM from three independent experiments. One-way ANOVA analysis, followed by Dunnett's test, was performed, with $*P \leq 0.05$, $**P \leq 0.01$, $***P \leq 0.001$ and ns = not significant.

C Mouse embryonic stem (mES) cells were treated for 3 h with 150 nM of LY411575, 1 μM of MLN4924, 10 μM of Roscovitine, 0.1 μM Purvalanol B or 10 μM RO-3306. DMSO served as vehicle control. Western blot analysis reveals that NICD levels were increased upon treatment with Roscovitine, Purvalanol B, RO-3306 and MLN4924. NICD is undetectable following LY411575 treatment. β-Actin served as loading control.

D HEK293 cells were treated with DMSO for 3 h. 10 to 100 μg of proteins was loaded. Endogenous levels if NICD were detected by Western blot. β-Actin has been used as loading control.

E iPS cells were treated with 0.1 μM of Purvalanol B for 3 h. Endogenous levels of NICD were detected by Western blot. β-Actin has been used as loading control.

F Quantification of the density of Western blot bands in (E) using ImageJ software. Data are expressed as fold changes compared to DMSO. All data represent the mean ± SEM from three independent experiments. Student's *t*-test analysis was performed, with $**P \leq 0.01$.

© 2019 The Authors

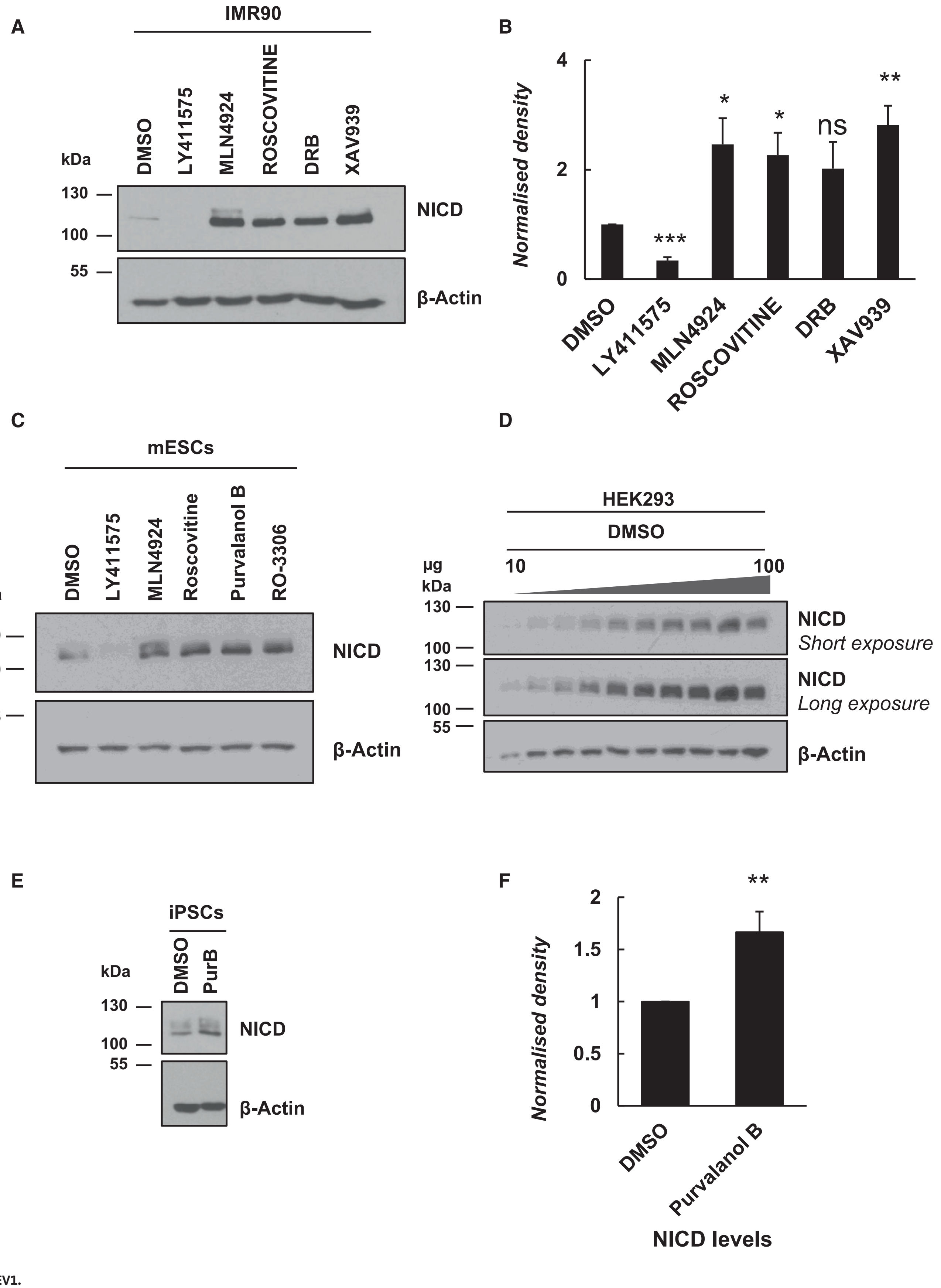

Figure EV1.

© 2019 The Authors

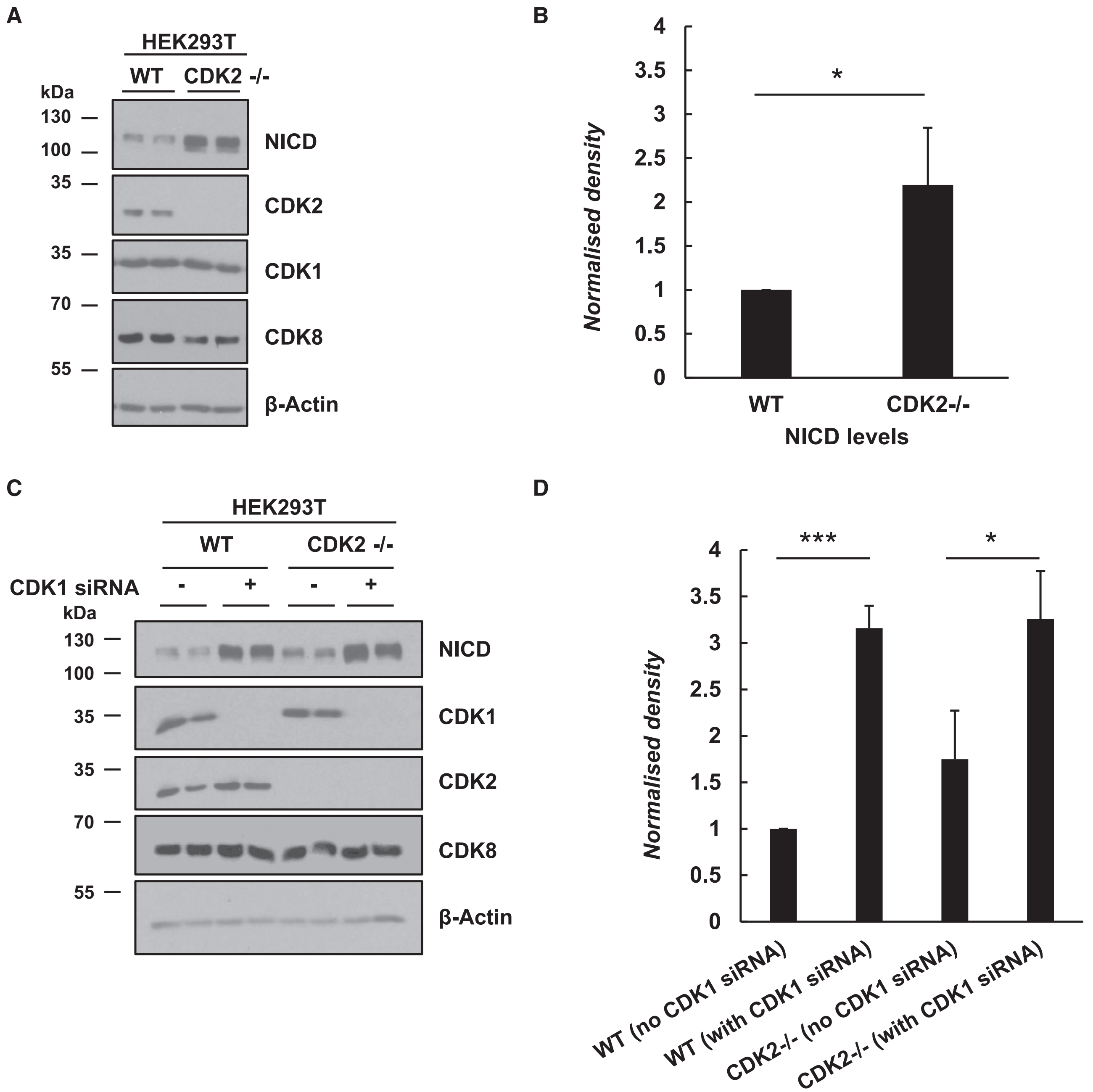

Figure EV2. CDK2 genetic depletion increased endogenous levels of NICD in HEK293T cells.

A CDK2$^{-/-}$ HEK293T cells, lacking endogenous CDK2, were cultured for 48 h after seeding followed by Western blot for NICD, CDK2, CDK1 and CDK8. β-Actin served as loading control.

B Quantification of the density of Western blot bands in (A) using ImageJ software. Data are expressed as fold changes compared to WT cells. All data represent the mean ± SEM from three independent experiments. Student's *t*-test analysis was performed, with $*P \leq 0.05$.

C CDK2$^{-/-}$ HEK293T cells were cultured for 48 h after transfection with plasmids encoding scrambled siRNA (−) or siRNA specific for CDK1 (+) followed by Western blot for NICD, CDK2, CDK1 and CDK8. β-Actin served as loading control.

D Quantification of the density of Western blot bands in (C) using ImageJ software. Data are expressed as fold changes comparing WT cells (−/+ CDK1 siRNA) and CDK2$^{-/-}$ cells (−/+ CDK1 siRNA). All data represent the mean ± SEM from three independent experiments. Student's *t*-test analysis was performed, with $*P \leq 0.05$ and $***P \leq 0.001$.

© 2019 The Authors

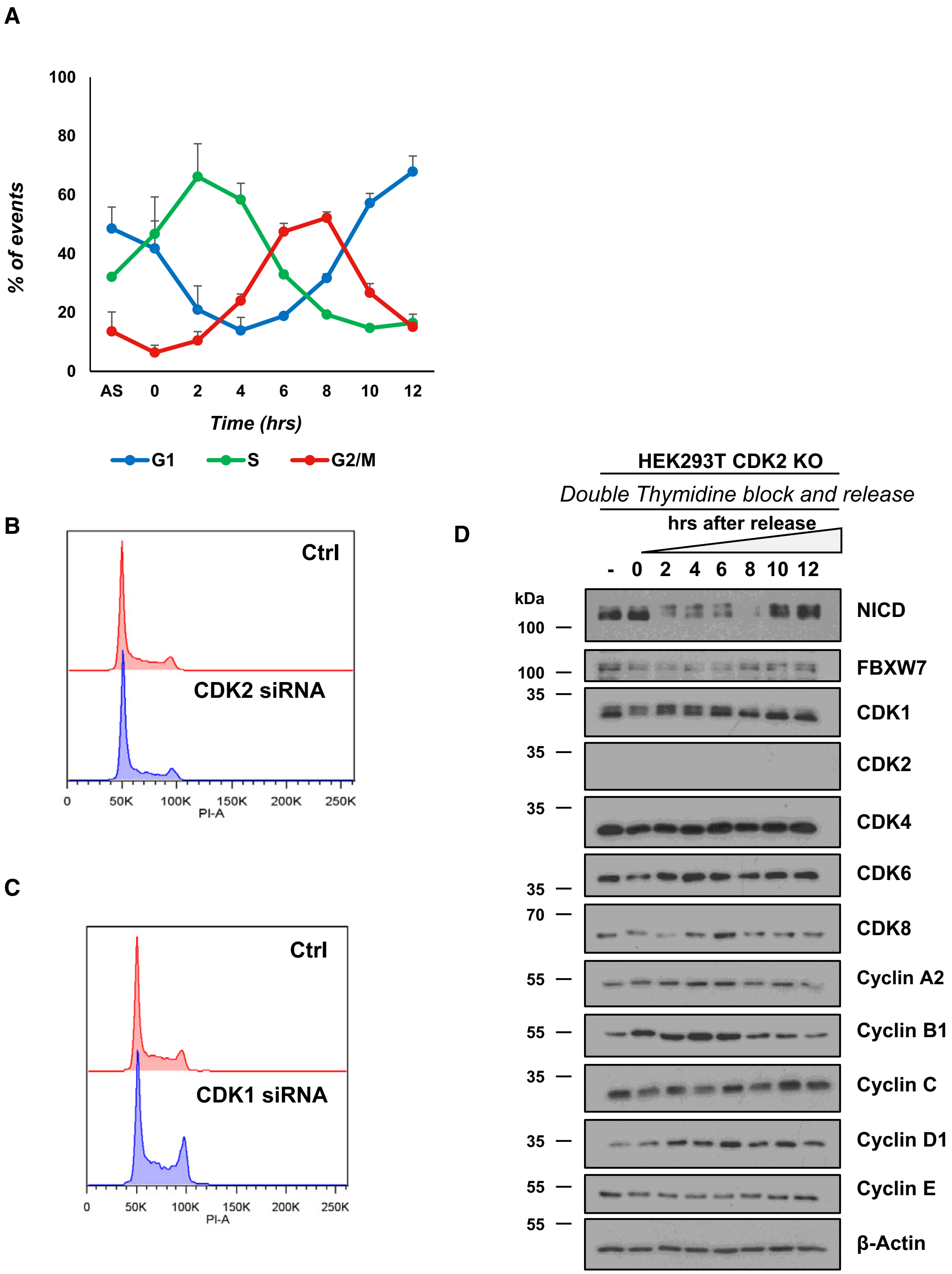

Figure EV3. Cell cycle profile of HEK293 cells upon CDK1 or CDK2 inhibition.

A Chart of flow cytometry data shows the percentage of HEK293 cells in G1, S and the G2/M phases after release from double thymidine treatment. Time points are expressed as mean ± SEM from three independent experiments.

B, C Cell cycle profile for HEK293 cells 48 h after transfection with plasmids encoding scrambled siRNA or siRNA specific for CDK2 (B) or CDK1 (C). Analysis of cell cycle arrest and release was performed using propidium iodide (PI) staining and flow cytometry. A representative experiment of three performed is shown.

D Expression of the indicated proteins in HEK293T CDK2 knockout (KO) cells after double thymidine block and release assay was examined by Western blotting, and β-actin was used as loading control. This summary is a representation of three independent experiments.

 © 2019 The Authors

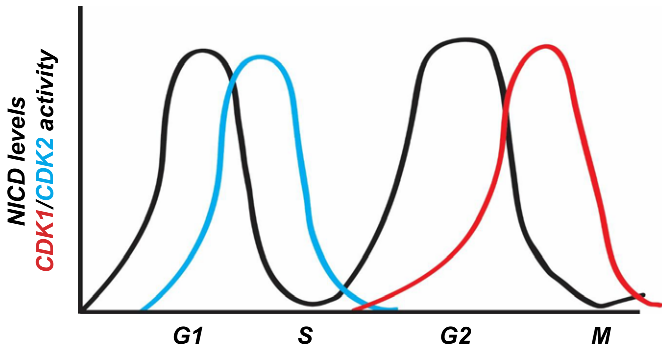

Figure EV4. Schematic representation of the link between levels of NICD, its phosphorylation and the cell cycle.

Levels of NICD (black line) fluctuate over the cell cycle phases (*x*-axis), and they inversely correlate with the activity of CDK1 (red line) and CDK2 (light blue line).

© 2019 The Authors
